# Elements of Operative Midwifery, Comprising a Description of Certain New and Improved Powers for Assisting Difficult and Dangerous Labours

**Published:** 1825-10-01

**Authors:** 


***Elements of Operative Midwifery, comprising a Description of certain new and improved Powers for assisting difficult and dangerous Labours; illustrated by Plates, with cautionary Strictures on the improper Use of Instruments.*** By DAVID D. DAVIS, M.D. Member of the Royal Colleges of Physicians of London and Edinburgh, formerly a Physician of the Sheffield General Infirmary; late Obstetric Physician to Her Royal Highness the Duchess of Kent; one of the Physicians to the Royal Maternity Charity for delivering

Poor Married Women at their own Habitations, &c. &c. Quarto, pp. 345. London, Hurst, Robinson, and Co. &c. June, 1825.

The numerous elementary and systematic works which have been published on midwifery since the time of Smellie, whatever they may have added as improvements to the practical precepts which he laid down, have absolutely failed in obtaining for this department of the healing art, a due degree of attention and respect from the profession in general. There are many enlightened and able men who, even while engaged in prosecuting the practice of other departments, with honor to themselves and advantage to the public, have not been ashamed to speak slightingly of midwifery, as a pursuit unworthy of a man of talents, and beneath the dignity of a science. Whether the little estimation in which midwifery has heretofore been held has been, in any degree, owing to the conduct of its professors, we shall not pretend to determine, but we believe that the time is fast approaching, when any gentleman pretending to have had a liberal professional education, will be ashamed, at least, to avow his ignorance of midwifery, and when the importance of precise and scientific acquaintance with its principles will be universally acknowledged by all, who can be considered competent to form a sound opinion on the subject.

Works on midwifery, as on other departments of medicine, have perhaps been obtruded upon the public, not so much for the purpose of conveying instruction or adding any thing new to the general stock of information, as to shew forth the merits and pretensions of some young candidates for practice. The volume of which the title is recited at the head of this article, is of a very different description. It is, in the strictest sense of the word, an original work, the fruit, evidently, of much laborious investigation and careful study, now offered to the profession as a contribution towards the improvement of that department to which, however unworthily despised by many, the learned author has long and zealously devoted his chief attention. The principal object of the work is to ascertain the principles upon which the expediency or necessity of resorting to the use of obstetric instruments ought to be determined, to point out the defects of the instruments heretofore in use, and to explain and recommend certain improvements in their construction and in the modes of operating with them, which the author's experience has suggested to him. From the little attention that has, for many years, been bestowed upon the study of this art, many of our readers may not be aware of the necessity of such a work, but we are sure that no person will deny that the subject requires investigation, if he will, for a moment, reflect upon the vast difference that exists as to the proportional frequency with which obstetric instruments are employed by different practitioners and in different obstetric institutions in various parts of Europe. To enable our readers to form a judgment upon this point, we shall here quote a statement from near the conclusion of the work under review, from which it appears that while, in some institutions, obstetric instruments have not been used above once in seven hundred and forty patients, there are individual practitioners who have actually used them as often as once in every three!

"Of all the questions that may occur during a deliberate consideration of this subject, (instrumental midwifery) none can exceed in importance that of the average frequency with which we should resort to the instrumental resources of our art. I am sorry to say that we are not yet in possession of sufficient documentary evidence to enable us to decide this point; whilst the evidence we have is of so unsatisfactory and conflicting a nature, as to afford us but very slender materials for useful practical induction. It has been stated by Professor Boer, (see Medicina Obstetrica, p. 443) that the forceps has been used in the practice of an individual, or of individuals, whom, however, he has not chosen to name, in *nearly* one case out of every three labours. Professor Hagen of Berlin (Versuch lines lehrgebaüdes der Pratischen Geburtshülfe) delivered thirty-nine women out of three hundred and fifty, or one in nine, with the forceps. Professor Nägele, of Heidelburg, reports, that in the practice of the Lying-in Institution, of that city, for the years 1817 and 1818, he used the forceps once in fifty-three cases; (see the Quarterly Journal of Foreign Medicine and Surgery, vol ii. p. 288) Mr. Burns (Principles of Midwifery, edit. 6, note on p. 441) gives the proportions of Professor Nägele as 'very much corresponding with those of his own list.' In a statement of presentations at la Maison d'Accouchemens, between December, 1799, and May, 1809, furnished by the late M. Baudeloque, (see Merriman's Synopsis, 306) we have the proportion of forceps to the whole number of labours, as one in three hundred and fifty-three. In a synoptic table of the practice of l'Hospice de la Maternité, between 1797 and 1812, giving all the labours at 20,357 during that period; Madame Boivin (see p. 354 of her Memorial de l'Art des Accouchemens) reports, that the forceps was used ninety-six times, *i. e.* once in two hundred and twelve cases. Madame la Chapelle, head Midwife of la Maison d'Accouchement of Paris, in her Pratique des Accouchemens, vol, i. table comparative, No. 3, reports the proportion of forceps cases to have been one to one hundred and sixty-six. The entire number of labours amounted to 15,481 in nine years, wanting a few weeks. I have private, but official authority for stating, that the forceps was applied eight times in the year 1823, in the lying-in wards of the Obstetric School of Göttingen; where the whole number of patients is stated to have been one hundred and fifty; giving the proportion of forceps operations to all the labours as one to eighteen and three fourths. I should in justice add, that my learned correspondent follows up this information with a very important explanatory remark; viz. that he is in the habit of exercising a large discretion in the selection, for admisssion to the institution, of persons most likely to require the assistance of art. In a similar communication from Dr. Cederschjold, Professor of Midwifery at the University of Stockholm, I have been obligingly informed, that the forceps had been used for some years past at the Obstetric Institution in that city, on the average of one in a hundred cases. Dr. Luders, a physician of the official rank of Royal Danish District Physician (Laud Physicus) at Eikenfüde, in Holstein, states the proportion of forceps cases, in his practice, as one in a hundred and nine, on the average practice of three years. M. Lobstein reports that he used the forceps, in the lying-in wards of the Civil Hospital, at Strasburg, twenty times in seven hundred and twelve cases (see Leroux' Journal de Médécine, vol. xxxvi. p. 125.) The late Dr. Bland, in his 'calculations of the number of accidents or deaths which happen in consequence of parturition', &c. &c. (Philosophical Transactions, vol. lxxi.) reports, that he used one branch of the forceps in four hundred and twenty-four cases. Taking the mean proportion of forceps cases as given in six different reports of the practice of the School of Midwifery, at Vienna, under the direction of the eminent Professor Böer, (vide Medicinæ Obstetricæ, p. 65, 131, 203, 429, 486, and 585) we have one forceps operation in two hundred and thirty-eight labours. In Dr. Clarke's Abstract of the Dublin Lying-in Hospital Registry, it is stated, that in the practice of that great national establishment, between the 1st of January, 1787, and the 1st of October, 1793, during which period 10,387 women were delivered, the forceps was used fourteen times.'

Dr. Davis considers the proportion of one in fifty-three, which is approved of by Professor Burns, as at least four hundred per cent too great, and he comes to the conclusion, that the forceps cannot be required more than once in three hundred, or, at most, once in two hundred and fifty cases. While such a striking difference of opinion exists upon a subject, the importance of which can hardly be questioned even by those by whom midwifery may be held in low estimation, it is impossible not to suppose that the use of obstetric instruments has heretofore depended upon the caprice or inclinations of individual practitioners, rather than upon the skilful application of any acknowledged scientific principles to the guidance of their decisions. A work, the object of which, is the ascertainment of such principles and the illustration of the rules according to which they ought to be applied in practice, cannot in these circumstances fail to attract the attention of the profession, and if it be executed with even moderate abilities, and contributes in any degree to remove the obscurity in which the subject has so long been involved, it must have a beneficial influence on the art. Whatever judgment may ultimately await the labours of Dr. Davis, it cannot be said that his time has been thrown away in cultivating a field where cultivation was not required; and, whether the opinions which he inculcates, and the alterations which he proposes, be generally adopted or not, the object he had in view must be more or less accomplished by the discussion of the subject. That he has taken immense pains to obtain a perfect knowledge of the art, the principles of which he professes to investigate and to teach, is manifested by the numerous and minute references which he gives to preceding authors. It is only upon reading the work carefully, however, that a just estimate can be formed of the extent of the author's reading, for although he gives accurate references for the illustrations which are furnished by other authors, he has not followed the usual custom of placing the references apart at the bottom of the pages so as to strike the eye. We have reason to know that in his search after information on this subject, Dr. Davis was not satisfied with all that his acquaintance with the ancient languages, and their more immediate offspring, the French and Italian, in addition to our own, were calculated to give him access to; but it deserves to be noticed, as no ordinary effort of assiduity, that he should have found it possible, within the last five or six years, in the midst of his otherwise laborious duties, as a public lecturer, and physician to several public institutions, besides the avocations of private practice, to make himself so far familiar with the German language, as to be able to read the works of the best authors, and to search throughout the numerous periodical works which are printed in that language, for facts illustrative of his favourite subject of study.

Extensive, however, as is evidently our author's acquaintance with whatever of value has been written upon the subject, it is impossible to peruse his work without being struck with the caution with which he admits either matters of fact or reasonings, which are not in some degree supported and rendered probable by concurrent facts, derived from his own experience. Indeed, it is manifest from his manner of treating the subject throughout the present work, that he has chosen to write about things with which he is practically familiar, rather than to display a specious and systematic classification of names which might have been wholly derived from books at infinitely less trouble and expence. On some important points of doctrine and practice, the learned author differs widely from opinions, which, having emanated at first from writers of great authority, have been received and acted upon by the profession in general almost without inquiry, and have been copied into minor systematic works as unquestionable axioms.

With respect to the instruments employed in midwifery, a glance at the plates will shew to any person who has paid attention to the subject, that some of the old ones have been re-modelled upon new principles, and that several entirely new, are proposed by the author to supply the place of the imperfect and unsafe instruments heretofore known to the profession. Our author, however, does not confine himself to the mere consideration of the mechanical questions involved in an inquiry into the best modes of constructing and using obstetric instruments, but enters in detail into all the circumstances that relate to the expediency of the operations themselves. In these interesting inquiries he draws pertinent and valuable illustrations from the works of preceding authors, as well as from his own observation. Though the work may, therefore, be said, in conformity with its title, to relate chiefly to the operative or mechanical part of the art of midwifery, it embraces, in fact, a full consideration of the most important questions of doctrine and practice belonging exclusively to this department of medicine.

Having premised these remarks on the general scope and character of this work, we shall now endeavour to give such an analysis of its contents as shall satisfy the readers of this Journal, that we have not over-rated its value, and at the same time, do justice to the new and interesting materials with which it abounds.

The work is divided into six sections, of which, the first is confined to general and introductory remarks on operative midwifery, and more especially on some of the causes that have contributed to retard its improvement as a branch of the Art of Medicine. As an explanation of the still barbarous and imperfect state of the operations of midwifery, Dr. Davis notices the fact, that except where the necessity of some surgical operation was anticipated, no male practitioner ever had been engaged to attend a female till one hundred and eighty years ago, when Julian Clement, a professor of surgery was employed to deliver the Duchesse de la Viliere, a mistress of Louis XIV. After this period, great obstacles existed to the general employment of male practitioners. One circumstance, which had great weight with the female sex, was the apprehension, by no means without some foundation, that instruments would be unnecessarily resorted to. After Gifford first used the forceps, some became zealous partisans in favour of that instrument, and the natural consequence was, that it was often indiscriminately and improperly made use of. Another party again were no less active in opposing and reprobating the forceps, and the same observations may be applied to the lever. These violent proceedings had the effect of involving the instruments themselves in discredit and neglect; or, in other words, of retarding professional improvement in this important department of practice.

"In adverting," says our author, "to the slow progress of improvement in the mechanics of midwifery in England, since the time of Gifford and Chapman, it is impossible to overlook the remarkable influence which was exerted in this respect by Dr. William Hunter. The substantial talents of that eminent individual, aided possibly by something of adventitious distinction to which he was raised by his contemporaries, gave great weight to all his opinions on popular and practical subjects. It unfortunately happened, that by virtue of his general reputation, he also exerted a predominant influence as a practitioner of midwifery, and most certainly without being justly entitled to the credit of pre-eminent qualifications for its duties. It has been, indeed, stated, that he professedly engaged in its practice as a source of emolument, and in perfect subservience to other objects of ambition. (See Dr. Foart Simmons' Life and Writings of Dr. William Hunter.) By a concurrence, however, of several fortunate circumstances, he soon succeeded to the practice of Sir Richard Manningham and Dr. Sandys, without the drudgery usually attendant upon what is called establishing a practice, and, therefore, presumably without having been compelled to make himself master of the greater difficulties of the art. Not to insist, however, on the value of this inference, it is a well known fact that he never made a single accession to its mechanical resources. It may be worthy of remark, that it was a part of the character of Dr. Hunter's mind to admire, even to devotion, the resources of unassisted nature, and to look with extreme jealousy and distrust upon all interferences of art, as auxiliary to natural functions. For these reasons, and others, perhaps, more immediately connected with the state of midwifery at his time, and having reference more especially to its then misfortunes, imperfections and competitions; he was not many years in the practice of it, before he became the leader of a powerful party of obstetric alarmists, who, by glowing representations of the sufficiency of nature in almost all cases whatever, and heavily charged descriptions of the abuses and dangers of instrumental methods of delivery, embodied a prodigious influence against the pretensions of operative midwifery in general;—an influence, indeed, which not only had the effect of repressing much of the ignorant daring of the period to which it was particularly intended to apply; but which has continued to operate with more or less activity ever since, and upon the whole, to the extreme prejudice, I might add, to the almost entire suspension of improvement in this department of the art."

Dr. Davis proceeds to notice the fact, that this department of midwifery seems not to have been made the subject of particular study by any person possessed of a turn for practical mechanics, either in this country or abroad, since the time of Smellie. The general body of practitioners have, indeed, wanted opportunities to prosecute it with advantage on a sufficiently extensive scale, while of the comparatively small number attached to the more extensive obstetric institutions, some may have neglected it from simple preference for other studies of perhaps equal importance to the art, without intending to disparage its value; whilst others, there is reason to fear, from a false estimate of its utility, imputable possibly to early prejudices, have treated it with blameable indifference, and, occasionally, as Dr. Davis believes, with loose levity and reprobation.

"Now," says he, "the natural function of parturition either does or does not, in any circumstances whatever, require the aid of instruments for its safe or even possible performance. If it does not, then in the name of all that is good and consistent in conduct, and for the sake of our common humanity, why does not some ascendant genius, some fond disciple of Hunter's wary school, shake off an unworthy lethargy in so sacred a cause, and advance boldly to proclaim the truth of so delightful a doctrine? But if on the other hand, it is an incontrovertible fact, that instruments are sometimes of great use in the practice of midwifery; nay, that they are sometimes indispensably necessary to save life; then, indeed, must it inevitably follow, that the business of this department of the art is at least as much the object of study and improvement as that of any other; and that to attempt to involve its very serious duties in discredit, or to connect with them the imputation of an inferior degree of importance, can only be deemed worthy of great ignorance or extravagant conceit—the sources, in fact, from which such conduct has invariably proceeded."

However strongly Dr. Hunter and his admirers may have insisted upon the propriety of abstaining from any artificial interference in the function of parturition, and upon leaving almost every thing to be accomplished by the unassisted powers of nature, we believe that no one in the present day will be found to defend the extent to which they carried their peculiar doctrines; for example, who will attempt to justify the dangerous practice which Dr. Hunter once adopted of leaving the placenta in all cases whatever to be expelled without manual assistance? Nor do we think that any person will be bold enough to accept Dr. Davis's challenge, and attempt to maintain that, the aid of instruments is not occasionally absolutely indispensable to the preservation of life. But, if this be conceded, the inference which he draws is not only logically, but naturally correct, namely, that this department has as good and legitimate a claim to be made the subject of study and improvement as any other; and, certainly, to hold it out as unworthy of serious attention can proceed only from culpable ignorance of its real importance. So far, indeed, is the importance of midwifery in general from being properly appreciated by the profession, that our author states it as

"A fact equally notorious and remarkable, that the profession of midwifery, for many years viewed with suspicion, perhaps with disdain, in certain high quarters, is not even yet deemed worthy of the smallest recognition, much less of protection and encouragement by any of the learned professional colleges or corporations of England. I am sorry to add, that those impressions, so obviously injurious to the best interests of the art, are not confined to the public bodies of the profession, nor to any privileged casts of practitioners, whether physicians or surgeons; who may be supposed to act under the influence of the jealous and exclusive spirit of their respective institutions. On the contrary, they prevail to a lamentable, and, I am afraid, to an increasing extent amongst the profession generally. I am informed, that of late years, it has become a very common practice with a certain class of students, whilst engaged in *walking* the hospitals, to speak of midwifery as a very subordinate object of attention, and of the knowledge of its 'few and simple duties' as being easily attainable by private reading, and therefore not worth the time and the very moderate expence usually bestowed on the acquisition of it by some of their better informed fellow-pupils. Loose notions of this kind, as of course they are not the original and innate conceptions of these young gentlemen's minds, nor can they be admitted to be the results of any legitimate and well-founded induction, are, no doubt to be ascribed to the influence of a very defective and vicious system of early professional education, to associations contracted during the listless years of apprenticeship, and not unfrequently, as I have good reason to know, to the ignorant counsels and precepts of incompetently educated masters. It is a fact, which it would be difficult to believe, were it not perfectly well authenticated, that these latter worthies have sometimes foisted into their last and farewell instructions to their pupils, when on the eve of departing for London or Edinburgh, to finish their professional education, the extraordinary advice, 'not to put themselves to much inconvenience as to attendance on midwifery lectures,' adding, 'that the principles of that art are few and simple, as also very well detailed in books; and as to skill in practice, that it is only to be acquired by actual experience on the living subject.' In accounting for conduct so preposterous on the part of these veterans (for I am not willing to impute it to any considerable number of the younger general practitioners of the kingdom,) we are bound to presume that they must themselves be miserably incompetent to encounter the greater difficulties of the art—often too ignorant even to recognize some of the difficulties here supposed, and therefore probably wholesale dealers in one or two of the more capital and fatal operations of midwifery. Such men, however, are many of your reputed clever fellows, the loudly lauded darlings of their districts, experienced masters of many arts, persons renowned for quick decisions, solitary and self-alone consulting operators, cool and steady-handed drivers of pointed steel into living fœtal sculls. Few and simple duties! What difficulties, indeed, could be anticipated, as likely to arise in the routine of ordinary practice, of sufficient magnitude to resist the power of such ruthless operators! Dead children, like dead men, can tell no tales; and at any event, still-births are occasional and unavoidable occurrences in the practice of the most consummate professors of the art. Nevertheless, let the wretchedly taught pupils of such instructors be persuaded at least to take the opinion of better informed advisers as to the essential value of obstetric studies."

Not being acquainted with the particular facts which Dr. Davis seems to have had in view when he penned the foregoing remarks, we are willing to suppose that there are but few practitioners extensively employed, to whom they could apply in their full extent, but we have inserted them here, because it is self evident that the preposterous custom of dissuading pupils from bestowing particular attention on the study of midwifery must have a tendency to produce practitioners, and to perpetuate practices such as Dr. Davis so warmly and justly reprobates. If there be any number of individuals in the profession to whom Dr. Davis's remarks might with justice be applied, we trust that in future, such men will be contented to pursue their practices in silence, and will desist from attempting to prevail upon young men to imitate their example by entering upon the practice of their profession without adequate preparation and study.

When we consider the erroneous notions that prevail so generally, among even the well-informed of our profession, respecting the importance of midwifery as an art, it is scarcely to be wondered at, that it should not as yet have been deemed worthy of any legislative protection. It would excite surprize, however, if, after the attention of the enlightened legislature of this country shall have been once directed to this subject, it should continue to be permitted to any set of persons to undertake the serious duties of midwifery without any legal responsibility, and without having undergone any examination whatever, as a test of their qualifications. Some well-digested regulations upon this subject might easily be devised, which, without doing violence to the prejudices of the fair subjects of the art, if any prejudices remain, could not fail to conduce to its rapid improvement, and are certainly necessary for the sake of establishing the distinction that ought to exist in law, as well as in reality, between practitioners who have been at the pains and expence of acquiring a competent knowledge of their profession, and those who do not scruple, for the sake of gain, to undertake the performance of its duties, while they ignorantly presume to characterize them as too unimportant to be made the subject of serious study or attention. In his remarks upon the prevalence of such mistaken notions upon this subject, Dr. Davis asks—

"Whether the instruments portrayed in this work, or any other powers of the same kind, and intended to answer similar purposes, are to be used, for the first time, and therefore, of course, by inexperienced and incompetent hands, upon the living persons, and living offspring of our fair country-women, or upon their lifeless representatives, in our theatres of midwifery, admirably calculated as some of these machines are known to be, to represent their living prototypes, and to furnish the pupil with the amplest means of qualifying himself for encountering the greater difficulties of the art in his after practice?"

He here takes occasion (as we think very justifiably) to allude more particularly to the extensive and unequalled apparatus in his own possession, which, from frequent inspection, and personal experience, we can safely affirm to be eminently calculated to enable the student to acquire dexterity and confidence in the most difficult and important obstetric operations which he may afterwards be called upon to perform in actual practice.

The habit that has so long prevailed of deprecating the use of instruments in the practice of midwifery, as rarely or never necessary, has probably had some influence in preventing improvements in their construction, from being proposed even by gentlemen fully competent to the task, from an apprehension that they might be represented as possessing an overweening fondness for the use of such powers. Dr. Davis appears to be aware of this danger; and it must in candour be allowed, that in venturing to brave it, he gives a proof of his sincere conviction that he does not bring his proposed alterations into discussion upon light or insufficient grounds, nor without confidence that they will stand the test of examination. He, however, deprecates any premature or hasty decision, and appeals to a numerous professional connexion, to attest that he is not remarkable for any predilection for instruments, in any case, where their use can be safely dispensed with. On this score, however, if the experience of others, who have been in the habit of consulting with him, and observing his practice, tallies with our own, he certainly has nothing to apprehend.

The second section embraces fully two-thirds of the whole work, and relates, first, to the varieties of modern forceps, and the modifications in their construction which the author proposes; secondly, to the numerous important practical questions which arise in considering the various circumstances that may indicate their use; and lastly, to the best mode of using them in every variety of circumstances in which they can be required.

"The objects of the art of midwifery," observes our author, "are to superintend, and, when necessary, to assist and direct nature in her efforts to accomplish the act of parturition, with all possible safety to the lives and living structures which are interested in the event. The process of child-birth being ordinarily the operation of a natural and a healthy function, is, for the most part, effected without much difficulty; so as very rarely to require the assistance, or to be benefitted by the use of artificial powers. To this established law, however, there are occasional exceptions, inasmuch as the function of parturition, like all other vital actions, is liable to morbid influences, and, perhaps, more than any other to mechanical obstructions and impediments. To assist nature in overcoming the difficulties which are casually produced by the operation of those agents, is properly the province of instrumental midwifery. When this object is attained compatibly with the preservation both of the mother and her offspring, and without inflicting a serious injury on either, then, indeed, is the triumph of our art complete. But the difficulties of some unfortunate cases are so great, and of such a nature, as to compel us to restrain our hopes of success within narrower limits, and to direct our principal, and, perhaps, our exclusive efforts to save one life, and preferably, if practicable, the more valuable life of the mother. Hence obstetric instruments have usually been distributed into two principal classes: the first consisting of two or three varieties of power; but possessing in common the competency of being used compatibly with the salvation both of the mother and her offspring: and the second including all cutting instruments and such as may be required to be made use of as auxiliary to cutting instruments, and, therefore, whenever unhappily resorted to, necessarily implicating either the certain destruction of life, as in embryotomy, or much risk of it, as in the case of the Cæsarean section."

Our author justly describes the curvature in the direction of the sides of the blades of the forceps first introduced by the celebrated Levret, and the shortening and tightening of the whole instrument by Smellie, as the greatest improvements that have been made upon the forceps, since its first invention with separate blades by the Chamberlins. He first states the objections to which the several forms of forceps at present in use are liable, and then describes the alterations which he proposes.

The French forceps is so narrow in its blades that no part of a full-grown fœtal head will engage in its fenestræ, as may easily be ascertained by the application of the instrument to a fœtal skull of the standard size, although we seldom meet with a French writer who does not presume upon and maintain the contrary. As the blades are each one fifth of an inch thick, the two together must occupy two fifths of an inch of the space within the pelvis, whenever the instrument is applied, which is an immense disadvantage in any case, where a difficult birth may be accompanied with any confinement of the maternal pelvis. The curve of the blades is a segment of a circle much larger than a fœtal head, and consequently the head, when embraced by the instrument, is only very partially in contact with the blades. This circumstance renders the forceps very liable to slip, to the great danger of the maternal parts, unless the head be firmly compressed, to the still greater danger of the life of the child.

The blades of the forceps bearing the names of Smellie, Denman, and Osborne, are all too narrow to allow any portion of the head to enter their fenestræ, and, consequently their thickness must always be added to the breadth of the child's head, and must occupy part of the space through which it has to pass. They are likewise liable to slip, from the narrowness of the blades, while, from the lock being situated immediately at the outlet of the vagina, the maternal parts are necessarily exposed to considerable risk of being pinched or contused when the blades are adjusted. The common short forceps, proposed by Dr. Davis, is contrived with the design of combining the advantages of all the others, and to obviate, as much as possible, the objections to which they are liable.

The author's description of his forceps could not be intelligible to our readers without the plates to which he refers in the course of it; but we shall here state, as briefly as we can, its characteristic qualities. The fenestræ are wide enough (one inch and a half at the widest part) to admit the parietal protuberance of the child's head to come into contact with the pelvis, and the curve of the blades is so adapted, as to apply closely to the child's head in every part of their inner surface. These qualities enable the practitioner to obtain a firm purchase from which the head cannot by possibility slip, while the blades are situated on parts of the head where they do not occupy any of the space through which its broadest part had to pass, and the power of extracting is obtained, without the necessity of making any degree of pressure dangerous to the child. In fact, with this instrument properly applied, the head is so completely embraced by every part of the blades, that considerable movements upwards, downwards, or rotatory, might be performed, if necessary, without the risk of the forceps slipping off the head, and with perfect safety to the maternal parts; and the head may be extracted without being exposed to more pressure than often occurs where no instrument is used. The curve of Levret, that in the direction of the edges of the blades, likewise possessed by this instrument, gives it a superiority over Dr. Haighton's forceps, which, as our obstetric readers will recollect, has only one curve, viz. that in the direction of their sides. On account of the want of Levret's curve, Dr. Haighton's forceps, which has wide fenestræ, cannot be used without more risk of lacerating the perineum than is liable to happen with forceps possessing Levret's curve. In a large majority of cases of operations with forceps, this instrument is to be preferred, on account of this curve, to the single curved forceps of Denman and Haighton, and for the remaining cases, viz. those with the face directed to either side of the pelvis, Dr. Davis proposes a variety of forceps upon an entirely new principle, a description of which we shall attempt to give when we come to speak of the cases for which they are designed. To obviate the risk of entangling the vagina in the lock of the instrument, Dr. Davis has lengthened the shank of his forceps, that is, the part comprehended between the lock and the fenestræ, so as to throw the lock nearly an inch and half further from the child's head, and, of course, from the external parts of the mother, than in any other English forceps.

As in England, or wherever the custom prevails of delivering women while lying on the left side, it may occasionally serve to facilitate the introduction of that blade of the forceps which applies to the upper or right side of the pelvis, to have the handle made to bend by a joint out of the way of the bed, Dr. Davis has given to his forceps a contrivance for this purpose, and likewise adverts, in terms of approbation, to a new joint for the same purpose, which has lately been adopted by Dr. Hamilton. By both of these contrivances, the blades are made to bend in the opposite direction to that to which the former hinge of the Edinburgh forceps was fitted to bend, which latter, instead of being an improvement, was really a disadvantage to the instrument.

Dr. Davis arranges, under five distinct heads, the circumstances indicating the use of the forceps, as follows.—

"1. The first and most important indication for the use of obstetric instruments of any kind, is a positive and well-ascertained insufficiency of the natural powers to accomplish the act of parturition with safety to the lives and structures implicated in the process.

"2. The use of the modern forceps presumes, also, upon a sufficiency of space within the pelvis to admit, with its assistance, of an eventually living birth.

"3. But even then, the forceps can only be used with propriety, when its application is, upon the whole, judged preferable to all other modes of delivery.

"4. Cases of head-presentations are generally considered, in this country, and I think justly, as the exclusive objects of treatment by this class of instruments.

"5. Inasmuch as the forceps cannot be used without exposing the mother to some degree of inconvenience, if not of positive injury of structure, it should never be employed for delivering dead children.

To determine when a deficiency of the natural powers has been evinced, in such a manner as to justify or demand the aid of instruments, constitutes one of the most important duties of an accomplished midwifer, to make use of a term occasionally employed by Dr. Davis, in the present work. He treats of the causes tending to produce this indication, in the same order in which they have usually been arranged by other practical writers, under two heads, viz. the want of sufficient action of the parturient organs: and the want of sufficient space to admit of the child being propelled through the pelvis, at the expence of the ordinary power exerted in the process. In discussing this, and the other circumstances connected with the use of the forceps in detail, our author has embodied the most approved principles of the art, as founded on acknowledged facts, and confirmed by his own experience, into a system of practical instructions, of which, as far as they are peculiar to the learned author, we shall give an outline along with some of the more valuable and interesting facts employed to illustrate them.

Dr. Davis is much disposed to doubt whether a want of sufficient power in the parturient organs ever occurs, except as an effect of exhaustion, or as an accompaniment of some important disease in the uterine system. The following truly practical remarks on the term exhaustion, seem well entitled to the notice of the profession.

"I know of no word in the language of midwifery that is more frequently misapplied than the term exhaustion, as used in connexion with cases of tedious and laborious parturition; and certainly of none in the use of which it is more important to be precisely and practically correct. It is, indeed, the constant language of the puerperal chamber, during every stage of a labour, with the exception of two or three of the first hours, that the patient is greatly exhausted; whilst, after the lapse of twelve or twenty hours, it often becomes a duty of no easy performance to the practitioner to support the spirits, and to calm the fears, both of the patient and her attendants. The practitioner must, however, obtain more substantial evidence for the fact of an exhausted state of the parturient powers than the mere sensations of the patient, or the fears of her attendants, to warrant his active interference.

"Exhaustion necessarily implies the previous possession of power. Experience proves that the efforts of labour are required to be exerted with extraordinary vigour, and for a long time, in order to produce a state of the agents of parturition even approaching to what might deserve the name of exhaustion. It is impossible to state any particular time for the accession, in any individual case, of such a condition of the constitutional powers as would properly admit the application of this epithet to it; as the fact could not be determined without reference, in every single case, to the previous strength of the woman, to the vigour and frequency of the parturient efforts, to the form of the pelvis, and to the condition of the soft parts concerned in the process, with many other circumstances. I do not remember having ever witnessed a state which I should consider myself correct in designating by the epithet exhaustion, presenting itself *within twenty-four hours* from the commencement of true labour pains. I do not mean to assert that the accession of other evils which might complicate and endanger the issues of the process, and which might require the interposition of artificial assistance, might not occur long before that period, but I am now speaking of exhaustion, from failure of the powers consequent upon previous and excessive exertion. After a duration, therefore, of only twenty-four hours labour, even though the action of the uterus might seem to be more or less perfectly suspended, it would be a novelty and, indeed, a perfect surprize to me, to find that such a suspension was the effect of exhaustion. On the contrary, I have known very safe and prosperous labours last for two and three entire days, without producing any thing like a state of exhaustion. The time occupied by a labour, is, therefore, never to be considered exclusively as a proper measure of its influence in the production of an exhausted state of the parturient powers.

"Again, I can have no idea of a state of exhaustion of the powers of parturition, in the case of a previously healthy subject, excepting in connexion with a considerable prostration of the general constitutional strength: whereas I am not very sure that such a condition can be presumed to exist, without its being accompanied by a dangerous contusion of the organs more immediately concerned in the labour."

Dr. Davis says he has never happened to witness a case of a puerperal patient dying undelivered, from a failure of the powers of parturition, as identified with the state of exhaustion in question, but he gives the following history, as exhibiting an assemblage of symptoms and appearances such as he thinks might be expected to be met with in cases of exhaustion.

"An unmarried woman, thirty years of age, had struggled with furious labour pains for several days. But they were now almost extinct, occurring only at distant intervals, feeble and powerless in their effect. The abdomen was prodigiously swelled, but, nevertheless, exquisitely tender to the touch, excepting in the immediate neighbourhood of the pelvis, where, the parts had probably lost a part of their sensibility, by the long and violent pressure to which they had been exposed. The pulse was small, irregular, gurgling, feeble, and beating one hundred and fifty strokes in a minute. The countenance was sunk and ghastly; but still retaining a glow of dingy red on the centre of the cheeks. The tongue was brown and dry. The temperature of the body scarcely exceeded the ordinary temperature of health; but the extremities were cold and clammy. The discharge from the vagina was extremely putrid and offensive. Using my index finger as a pelvimeter, I ascertained the measure of the conjugate diameter of the brim of the pelvis to be at least two inches and three quarters, and that of the lateral diameter to be upwards of four inches. The dimensions of the outlet I considered to be such as not to require any precise admeasurement. The orifice of the uterus was fully dilated, and the head of the child loose and flabby, and, with scarcely any protrusion of its integument, rested (where it probably had done for forty or fifty hours) upon the brim of the pelvis."

In this state of the patient, preparations had been made for performing the Cæsarean operation, and if that operation were never to be had recourse to till the natural powers have become exhausted, as in this case, it would, indeed, be suprising if any result were to follow but the fatal one which has uniformly, we believe, except in one instance, attended its performance in this country. In the present case, some doubts of the necessity of the operation having arisen, Dr. Davis was called in. He effected the delivery, with very little difficulty, by the operation usually termed cephalotomy. The patient appeared to rally for some hours afterwards, but there was no substantial improvement of the most formidable symptoms. The pulse improved in power without becoming less frequent. The abdomen gradually became less sensible to pain when pressed, but increased rapidly in distension. During the last two days of her life, she was harrassed with constant hiccups and vomiting, and about eighty hours after her delivery she expired. The cuticle of the child furnished evidence that it had been dead many hours before the delivery.

"On opening the body after death, the viscera were seen floating in a considerable quantity of turbid fluid, of the consistence of thin pus, extremely offensive as to its fetor, and of a darkish gray colour. The omentum was of the same colour, and perfectly putrid throughout its entire structure. The posterior chamber of the pelvis was full of the same kind of putrid fluid. The peritoneal lining of the whole abdominal cavity, including also the coverings of the uterus and other viscera, presented the appearances of different stages of mortified animal structure. The middle and inferior parts of the womb, more especially those which had been most exposed to compression during labour, were of a grayish black aspect, resembling very perfectly the colour of plumbago. No part of that organ had suffered solution of continuity. The omentum tore into shreds upon raising the anterior covering of the abdomen.

"The actual dimensions of the pelvis, which is in my possession, were accurately measured, and produced the following results:—the conjugate diameter of the brim was three inches; the transverse diameter of the brim four inches and seven eighths: both oblique diameters were precisely four inches and a half. The conjugate diameter of the brim was the shortest line which could be drawn through the center of the superior aperture, between any two points of its circumference; the long or antero-posterior diameter of the outlet was four inches and a half, whilst the short or transverse diameter was three inches and three quarters. The sacrum was not defective as to its concavity.

"Now, had the Cæsarian operation been indicated in the above case, which it most unquestionably was not, it should have been performed at an early period of the labour, and not after the expiration of three whole days of suffering, when it was next to a positive certainty that the child was dead from severe and long-continued action of the uterus upon it; and when that organ itself, and the other parts more immediately concerned in the struggle, were, in all probability, in a state of incipient gangrene. But considerations of this sort will come to be discussed with more propriety hereafter. The case is brought forward here, as the subject of such an assemblage of symptoms and post-mortem appearances as, I believe, would always be found to accompany that particular state of the parturient function, properly called exhaustion. I cannot, indeed, conceive any kind of principle inherent in these organs, which can be supposed to be capable of arbitrary fulness and exhaustion, independent of certain corresponding states of the organs themselves. These important agents, it is true, are liable to morbid actions, either immediately on account of diseases of their own structure, or indirectly, in consequence of being enfeebled and oppressed by the results of diseased actions in other parts. As to the proper indications in cases of this kind, I shall presently have to consider them at some length. I wish now to confine the reader's attention to the subject of exhaustion of healthy powers possessed, at the commencement of a labour, by a healthy subject: and I beg to repeat that, in such circumstances, my opinion unequivocally is, that a state of exhaustion is to be identified with a state of so much contusion of parts, as to involve the eventual results of a labour in great doubt and danger. If this be a correct view of the subject, what must become of the rule which some authors have laid down with extraordinary confidence, 'that instruments should never be had recourse to until indicated by a state of positive exhaustion of the natural powers, as the only sufficient evidence of their necessity!' It is obviously founded either on an abuse of words, or upon false and dangerous principles: and, in either case, it cannot be too soon discarded from our list of indications."

We have chosen to give these remarks of the author upon exhaustion in his own words, without attempting to curtail them, because it is a subject on which his opinion is apparently at variance with one that has been the current doctrine of teachers and writers on midwifery, in this country at least, for many years.

As one of the causes of deficient action of the powers of parturition, Dr. Davis treats of organic diseases of the organs concerned. Under this head he gives some interesting cases in illustration, of which the following is one.

"The subject was a patient of the Northern Dispensary. She presented her subscriber's letter fourteen or fifteen weeks before her expected time of confinement. She had had several children, and was now in the forty-fifth year of her age. Her visage was pale, furrowed and anxious; and she was manifestly suffering from some important disease. She had been the subject of frequent menstrual appearances, as she herself believed, from an early period of her pregnancy. The protuberant part of the abdomen was considerably tender upon pressure: the functions of the bladder and rectum were greatly disturbed, and seldom performed without occasioning much distress: the pulse was frequent and feeble. On examination per vaginam, I found the anterior lip of the orifice of the uterus, its anterior parietes generally, as far as the finger could reach, as well as the contiguous parts of the bladder, in a state of extreme sensibility and induration. The orifice of the wound was imperfectly closed. The patient was herself confident that she was pregnant. The uterus was decidedly in a state of considerable development; but as I could not feel any part of the child through its parietes, I abstained from giving, or even forming an ultimate opinion as to the nature of its contents. The poor woman had not, indeed, mistaken her case: for at the full period of her gestation, and very punctually to her reckoning, her labour unequivocally declared itself. When she had been about eight hours engaged in the struggle, I was summoned to the assistance of the gentleman whom I had appointed to attend her. The orifice of the uterus was then dilated to about two inches in its lateral diameter, but, upon the whole, very unequally; as the anterior part of its structure remained of a gristly hardness, and exquisitely sensible to the touch. In this state of things, it was deemed prudent to wait a few hours for the full effect of dilation of such parts of the orifice and neck of the womb as were not diseased. In the mean time the labour made tolerable progress: the head of the child gradually descended into the cavity of the pelvis; but the distress, or rather the anguish of the miserable patient, increased in a tenfold degree as the business advanced, from the increasing pressure of the fœtal head against the diseased parts in front of that cavity; and the scene altogether was truly pitiable. In about sixteen hours from the commencement of the labour, the pains became extremely feeble and unproductive. The constitutional powers of the patient having suffered great reduction of strength from repeated, and some considerable losses of blood during the latter weeks of her pregnancy, it appeared, at length, more than probable, that nature would not eventually be able to accomplish her object—provided it were safe and feasible to surrender a poor creature, already so much afflicted, to the further tortures of such a hopeless effort. I accordingly passed up, on either side of the head, the counterparts of a narrow bladed forceps; and with the utmost tenderness, and without difficulty, effected the delivery. The child was of the usual dimensions at the full period of gestation; but extremely meagre and flaccid as to the softer materials of its structure; it was, moreover, still-born; and from the easy abrasion of its cuticle, it had probably died in consequence of a hæmorrhage which the mother had sustained about three days before the accession of her labour, and since which she declared that she never felt it move. After this melancholy confinement, the poor woman never recovered sufficient strength to be able to leave her bed. She lingered, however, the victim of more than ordinary suffering, even from cancer, for a period of about sixteen weeks after her delivery.

"On inspecting the body after death, the contents of the hypogastric region of the abdomen, including large portions of all the structures there situated, intestines, bladder and uterus, were found matted together into a horrible mass of cancerous disease."

For a parallel case to the above, Dr. Davis refers to the Journal de Médecine, Chirurgie et Pharmacie (Corvisart's) vol. v. p. 304. Some time after the accession of labour, the pains suddenly ceased, and the patient died. On the inspection of the body, a short time afterwards, the uterus was found extensively diseased and ruptured, the child having escaped into the cavity of the abdomen.

Our author goes on to state that the uterus may suffer a great diminution of its parturient power from diseases affecting its own structure, or that of organs immediately subservient or contiguous to it, which might not in themselves be necessarily fatal in their termination. In illustration of this opinion, he relates a very interesting case of a painful tumour situated high up in the left iliac region, which terminated in a discharge of pus from the vagina. About fifteen hours after the commencement of labour the pains wholly ceased, and continued suspended for thirty-six hours at first without any circumstance besides to excite any alarm, but at the end of that period, the countenance had become anxious and somewhat shrunk: the pulse had risen to a hundred and ten: the stroke of the artery had become quick and sharp: the patient had slept none: the tongue was rather loaded, and vomiting had more than once taken place: the orifice of the uterus and the vagina were devoid of moisture. The patient was bled freely, and a brisk purgative was ordered, and as the woman was the mother of several fine children, and had formerly had easy and prosperous labours, Dr. D. resolved to wait some time longer. Late in the evening, he was called to the patient, she having been harrassed with violent vomitings. Her countenance was now expressive of deep anxiety: pulse much accelerated and irregular: abdomen uniformly painful to the touch and tympanitic: the patient vomited every five minutes: there was no sensible alteration in the state of the iliac tumour: the atmosphere of the room was now, for the first time charged with putrid fetor proceeding from a mixture of meconium and grumous blood which was issuing copiously from the uterus: the orifice of the uterus was remarkably soft and limber; the head of the child was presenting at the brim of the pelvis, with its vertex towards the left acetabulum; the liquor amnii had escaped at an early period of the labour. Dr. Davis applied the long forceps, and easily succeeded in bringing the head into the pelvis, when the uterus remaining torpid, he deemed it advisable to finish the delivery. The child was still-born, the cuticle peeling off with the slightest touch. The placenta soon came away in a very putrid state upon the slightest pulling at the cord, and was accompanied with a putrid coagulum of blood, but no hæmorrhage succeeded. The uterus was felt to have contracted, but not so much as in a healthy labour. The adventitious tumour in the left iliac region had sunk lower down but continued exquisitely painful. Some hours after the delivery, the patient was attacked with a smart symptomatic fever, which continued in a greater or less degree, notwithstanding such prompt and active treatment as the symptoms required, till the twenty-seventh day after her delivery, when, as already mentioned, there suddenly issued from the vagina a profuse discharge of pus. After this event the tumour was no longer to be felt in the iliac region, and the patient gradually recovered a state of moderately good health.

Dr. Davis's very judicious remarks on this remarkable case we shall give in his own words.

"Whether the iliac tumour was originally a disease of the uterus, or of the left ovary, and other contiguous parts, I am really not able to decide; for it left no cicatrix, induration, nor other evidence of its existence on any part of the uterus accessible to examination. I feel myself still less competent to account for its extraordinary influence in occasioning so sudden a suspension, amounting in fact to a complete extinction of the parturient faculty. The cause of the child's death was, probably, the premature separation of a considerable part of the placenta from the uterine surface. That was, no doubt, also the source of the putrid discharge, which, with other alarming symptoms, became finally decisive of the expediency of artificial aid. Whether that incident was proximately connected with the diseased tumour, can only be assumed as a matter more or less of probability. In the absence of external hæmorrhage, or any other evidence that such an incident had occurred, it is obvious that no specific time could have been chosen for the extension of artificial assistance, of which the choice would have ensured the preservation of the child's life: and I hold, that in reference to the use of instruments, it is a much safer principle not to act at all, than to act without the guide of a distinct indication. It is, indeed, possible that the life of the child might have been preserved, had the forceps been applied, and the delivery effected within a quarter of an hour after the suspension of the uterine contractions. But is there an experienced practitioner in Europe who would consider himself warranted in having recourse to the use of instruments in the circumstances of the above case at so early a period of the labour?"

Our author also gives the details of a case of a painful affection of the bladder, which was attended by a total suspension of the labour pains, and was followed in the third week after delivery, by a discharge of pus through the urethra. The case is well entitled to the attention of the profession in general, and more especially to that of practitioners of midwifery, who cannot fail, if engaged in extensive practice, perceiving the value of such cases, and of the truly practical remarks with which they are accompanied. A practitioner whose mind is well stored with practical facts, such as these cases are calculated to impress upon the memory, can often avail himself of such knowledge, and he is most likely to feel the value of it, when called upon to form a diagnosis in cases of difficulty, or to form an estimate of their probable duration and results. The greater his assiduity and success has been in acquiring and digesting this species of knowledge, the greater in all human probability will be his success in practice, and the eminence to which he shall attain in his profession. A work which abounds with cases like these, illustrative of the various subjects of which the author professes to treat, is far better calculated to promote the improvement of any department of our art than the most elaborate essays or finished reasonings without the detail of facts. Such a work on midwifery, is that now under examination, and we have no doubt, even the best informed in the profession, will not rise from its perusal, without feeling that they have made an acquisition to their professional knowledge.

To return, however, from this digression, we have next to recommend to the attention of our readers, the excellent remarks of our author on dropsy of the amnion, considered as a cause of deficient parturient action, which, like twin births, may sometimes produce this effect by over distension of the uterus. On the latter description of cases, when considered under the head of deficiency of parturient power, our author closes his observations as follows.—

"Under proper management of the constitutional powers, twin births are for the most part very safely accomplished without the assistance of instruments. On the other hand, cases of difficulty from this cause, admit of a very beneficial extension of artificial aid, at less expence of risk from its application than almost any other. The children of twin births are generally under the full size, and therefore more easily withdrawn through a passage of ordinary dimensions than those of standard growth; and, therefore, obviously with less chance of injury to the important structures within and at the outlet of the pelvis. The soft parts, which are most especially liable to contusion in these cases,—not from instruments, indeed, but from the natural agents of parturition, are generally those which are attached to, or situated in the immediate neighbourhood of the brim of the pelvis. In one of our indications for the use of the forceps, it was stated 'that head presentations are generally considered in this country, and in my opinion properly, as the exclusive objects of treatment by this class of instruments.' The principle must be fully recognized in its application to twin births. The general indication for an eventual appeal to this variety of artificial power must, of course, in this, as in all other cases, have a direct reference to its positive expediency. The elements of a proper estimate of such expediency will be found in a due consideration of the conditions of the soft parts, as to dilatation, irritation, and threatened danger of contusion or laceration, that of the accessibleness of the fœtal head or heads; the duration and actual character of the inertion, or incompetent action of the uterus; the state of the constitutional powers, and the occurrence of dangerous incidents, to complicate the labour."

In treating of cases where the action of the uterus is deficient without any obvious cause, our author makes some observations on the pretensions of those remedies which have from time to time been recommended as possessing a specific power of exciting the dormant uterus to action. He dismisses this subject with the following pertinent remarks on the ergot of rye, the remedy whose claim to such a power has most lately been urged upon the notice of the profession.

"If the ergot of rye or any thing else, which might be safely administered to a woman in labour, through the medium of the stomach, could be really proved to possess, in any considerable degree, a parturient influence on the uterus, it is obvious, that it would form a most important addition to our materia medica, as it could not fail, in a certain class and proportion of cases, to supersede the use of mechanical instruments in the practice of midwifery. The pretensions, however, of the secale cornutum have been generally known to the profession for a period of nearly twenty years, and yet the actual fact of its power has not been satisfactorily established, nor is there any evidence that I am acquainted with, that it has, in any one instance, superseded the necessity of using the forceps."

Our author now proceeds to make some observations on deficient action of the uterus from general constitutional weakness, and on the influence of diseases, or the results of diseased states of distant organs, as causes of deficient action of the uterus. In the course of his remarks under the last head, he mentions that he has seen four cases of emphysema from the rupture of a part of the bronchial structure of the lungs during labour, an incident which he thinks imputable solely to extreme severity of the labour pains. In all the four cases which he has seen, the emphysema occurred in the midst of a tremendous excitement of the heart and arteries. In every one of them the most marked relief was afforded by very copious bleeding. Three of the patients were delivered without mechanical assistance, and in the fourth, the forceps was introduced to improve the position of the child's head, and then withdrawn. The children were all born alive, and the mothers recovered satisfactorily, the emphysema rapidly vanishing without puncturing the integuments.

With these observations our author concludes what he has to say relative to deficiency of the natural powers as an indication for the use of the forceps, and proceeds to the consideration of another description of difficult labours, viz. those dependent upon defective capacity of the parturient passage.

"Difficult parturition," says he, "from defective capacity of the parturient passage, may arise from rigidity, or other unfavourable or morbid conditions of the soft parts, or from original confinement, or vicious conformation of the bones of the pelvis."

Besides these, our author here treats of such causes of difficulty as may occasion, or be attended with, a want of due proportion between a passage of ordinary amplitude and the bulk of the child or children to be propelled through it.

Our author's observations on difficult parturition from rigidity and other unfavourable states of the orifice of the uterus, such as coalescence of labia, &c. are well entitled to particular attention. They abound with valuable facts, illustrative of the duties of the medical attendant, and with ample and detailed references to the best sources of information on the subject. Without altogether proscribing, he does not think so favourably as Professor Burns seems recently to have done, (see the sixth edition of the Principles of Midwifery, p. 417—419) of the practice of assisting the dilatation of the orifice of the uterus with the fingers: and alludes to a case where that practice was followed, on the third day after delivery, by a fatal hæmorrhage, in which it was found, on inspection after death, that the os uteri was in a state of sphacelus. He does not see any good reason for bleeding prospectively, or during pregnancy, in anticipation of a rigidity which might or might not be realized by the event, although this practice was proposed by Dr. Rush, and has since been carried to a great extent by other American practitioners of eminence.

"To say the least of such a practice," says our author, "it would appear to be a most unnecessary encroachment upon the ordinary dispositions of nature, in the affairs of a function which she usually performs very safely and satisfactorily without any such interference. Bleeding, on the other hand, as a remedy corrective of an actual existing rigidity of the soft parts, whether or not accompanied by more than ordinary constitutional excitement, is a power of great and unquestionable value; and we are indebted to our trans-atlantic brethren for much useful discussion of the subject, and more especially for pioneering us through a wilderness of prejudices, into a just estimate of the extent to which, in certain imperious circumstances, it may be safely carried. In cases of protracted labours from rigidity of the soft parts, and accompanied by much suffering, and great excitement in the heart and arteries, I have often directed the abstraction of between thirty and forty ounces of blood, with the most marked advantage. I am, indeed, perfectly well convinced that, in cases of tediousness and difficulty from the causes here supposed, this important measure, well-timed, well applied to its objects, and judiciously combined with other means, which it would be improper to neglect, will not unfrequently have the effect of superseding the necessity of using instruments, when, otherwise, their employment would become indispensable. I could also refer to a variety of instances where it has enabled me to apply the forceps with the most perfect success in all respects, many hours earlier than, without its adoption, it could have been a safe measure even to make the attempt."

The following remarkable case of a contracted vagina will be read with interest by practical accoucheurs:

"I was once consulted in the case of a very young woman, in her first labour, the superior part of whose vagina was so contracted, that I found it impracticable to pass the point of my index finger through the part affected, in order to feel the orifice of the uterus. The spastic ring (for I believe that was the actual nature of the case) was situated as near the orifice of the uterus as I could suppose it possible for such an affection to exist. The feel of the part was that of a firm rugose texture, gradually puckering from some distance below, but, in no degree, giving the impression of a scirrhous hardness. The patient was, at the full period of her gestation. Her pains were regular, and decidedly parturient. She had been in labour about eighteen hours; and there was now developed a considerable excitement of the heart and arteries, accompanied by a state of more than ordinary irritation. After deliberating a short time upon the actual and prospective circumstances of the case, I directed venesection ad deliquium animi: and that effect was obtained by the abstraction of about thirty ounces of blood. Immediately after, a draught, with sixty drops of Battley's liquor opii sedativus was administered. I then left my patient, with an intimation to the gentleman in attendance that I should call again in a few hours. In about five hours afterwards, I accordingly did call, and found that the child's head had completely cleared, not only the orifice of the uterus, but also the contracted part of the vagina, and was beginning to bear upon the os externum; and in less than eight hours from the bleeding, the patient was safely delivered of a fine living child."

The subject of rigidity, contractions, adhesions, &c. of the vagina, is concluded with numerous and accurate references to the best sources of information, for which any gentleman wishing to study the subject minutely will feel thankful to the learned author.

The next part of his subject of which he treats, is the defective capacity of the parturient passage, from tumours within the pelvis. Some of these tumours are excrescences from the orifice of the uterus, and surface of the vagina; others are identified more or less intimately with the proper structures of these organs; and a third are situated within the pelvis, but externally to the vaginal and uterine parietes. According to our author, those of the first class might generally be extirpated with safety during pregnancy, by ligature or excision. He is of opinion, that no part of the parent structure of the fungus should be included in the ligature, or otherwise made the subject of operative violence, and, in support of this rule, he quotes a fatal case, communicated by Mr. Fordham, and published in the 26th volume of the London Medical and Physical Journal, page 38. When, from the broadness of the base of such tumours, or from other causes, it is deemed not advisable to attempt their extirpation before labour, and where it would be possible to deliver by turning, our author lays it down as a practicable and better method to deliver with the forceps, recommending instruments especially adapted for such cases, for the views and descriptions of which, the work and the plates must be consulted.

A large proportion of tumours of the third class in Dr. Davis's arrangement have been morbid enlargements of the ovaries. In some cases, where the contents of the tumour are fluid, and its bulk not incompatible with the passage of a living child, it may be proper to leave the case to the unassisted operations of nature. For others, several modes of relief have been proposed, and are respectively discussed with Dr. Davis's usual ability, viz. the Cæsarean operation, embryotomy, incision or puncture into the tumour, with a view to emptying its contents, and extirpation by an opening through the abdominal parietes and into the peritoneal cavity. Of the first and last of these operations for such cases, Dr. Davis expresses his unqualified disapprobation. Perhaps, however, when he shall have seen the cases of gastrotomy lately published by Mr. Lizars, he may see reason to modify his opinions in some degree. Further experience is necessary before these operations, which Mr. Lizars has shewn not to be necessarily fatal, are pronounced utterly unfit to be repeated in any circumstances. Embryotomy cannot always, in such cases, be performed with perfect safety to the mother, but the cases of extra-vaginal tumours more especially indicating it, are those where the tumour is either known or strongly suspected to be a hernial protrusion of intestine: or cases where the operation might be performed without much risk of increasing the present danger, or compromising the future interests of the mother: or, lastly, cases where a puncture or incision into the tumour might have been made without being followed by a sufficient diminution of its bulk to permit the passage of a living child. Our author's critical review of the numerous cases of this description which have been published by Mr. Perfect, Dr. Denman, Mr. Park, Dr. Merriman, and others, will be found by practical accoucheurs to throw a flood of light on this truly difficult subject, and to make up, in a great measure, to young practitioners for the want of personal experience; at least, a careful attention bestowed upon our author's perspicuous observations, and a perusal of the cases to which he has, with his usual precision, taken the pains to give accurate references, will enable any intelligent practitioner to discriminate such cases when they occur, and to apply, with confidence, the most appropriate measures of relief.

His Observations on Hernia of the Bladder, and on Over-Distension and Misplacement of that Organ, as interfering with the Function of Parturition, are also well entitled to attention. With this subject, our author concludes what he has to say respecting defective capacity of the parturient passage, as depending on affections of the soft parts; and now proceeds to treat of defective capacity of the pelvis, and the degree of confinement, admitting the safe use of the forceps. We shall only extract the following part of what he says upon this subject, recommending the whole, however, to the attention of our readers.

"If, indeed, it be a fact, that the natural agents of parturition are equal to the production of as much compression of the fœtal head as it is really capable of sustaining with impunity to the soundness of its organization, and more than this has been often proved; then the only question that can arise, as to the expediency of having recourse to the use of forceps in difficult births from this cause, must have for its object to determine how far the prompt assistance to be obtained from the application of artificial force might be presumed to deserve a preference, in some special cases, to a deliberate surrender of the issues of a hard labour to the unaided operation of the natural powers? Or, in other words, whether, in some peculiar circumstances, it might not be found more eligible to apply to the fœtal head a certain required amount of compressing force, within a short space of time, artificially by means of the forceps, than to await the result of a more gradual and protracted application of an ultimately equal degree of force by the natural agents of parturition.

"If it were possible, by artificial means, to apply to the fœtal head the compressing force here supposed necessary, without also, at the same time, exposing the parts situated within the pelvis of the mother to a degree of pressure which might have the effect of producing a dangerous contusion of their structure, I should not feel much difficulty in answering this question, upon the whole, favourably to the pretensions of operative midwifery. Assured, however, as I am, that in the circumstances supposed, which I assume to be those of defective capacity of the maternal pelvis in more than one of its diameters, it would be generally impossible to effect the good here contemplated, without also incurring more or less of the evil incident to such an attempt, I feel it my duty to express my opinion, conclusively, as unfavourable to the general principle of having recourse to the use of the forceps in cases of difficult births, from defective capacity of the maternal pelvis."

The exceptions to this general rule, with the proper methods of employing the aid of the forceps where it can be beneficially employed in confinement of the pelvis, are distinctly pointed out under the present head, although more fully illustrated by the learned author when he comes to treat of the long forceps.

The next subject which Dr. Davis treats of as connected with the use of the forceps, is arterial and constitutional excitement during labour. While an accelerated state of the circulation is a common effect of long continued parturient efforts, and may be perfectly compatible with the safety of the patient, yet, at the end of a severe and protracted labour, great vascular excitement may be a symptom of dangerous inflammation from contusion of the parts concerned. Delirium supervening during labour is an evidence of more than ordinary excitement of the vascular system, and of a dangerous fulness of the blood-vessels of the head, shewing a great tendency to convulsions. Delivery by the forceps, in such cases, the learned author is of opinion, should never be attempted without being preceded by general bleeding on a liberal scale. He says that bleeding to between twenty and thirty ounces, according to the special exigency of the case, will not only operate as a means of immediate relief to the patient, but will also prevent the accession of convulsions, and alter the whole character of the labour, so as eventually to supersede the necessity of resorting to the use of instruments. In support of this opinion, Dr. Davis states that he has never met with a case of delirium in his own private practice, a fact which he attributes to his having never suffered constitutional irritation and excitement to accumulate so far, at any one period of labour, as to expose his patients to the danger of delirium.

"But bleeding," says he, "our greatest agent of active practice in such a case, may come too late; or, from the magnitude of the obstacles to delivery, it may not itself be able to furnish a sufficient protection against all the evils, present and future, incident to severe labours. It will, however, seldom fail to be the harbinger, at least, of a temporary calm after a violent storm of suffering; which, at all events, will give to Nature further time to refresh and to concentrate her powers, and, to the practitioner, an opportunity to deliberate coolly on the actual position and prospects of his case, as also on the expediency and choice of ulterior measures. I think I may safely say that, in the majority of such cases as we are now supposing, a free bleeding will have the effect of manifestly improving the parturient efforts of the uterus, both as to their power and regularity; that it will produce an increased secretion of the proper mucus of the passage; that it will lower the temperature, and diminish the tenderness and swelling of the parts within the pelvis most exposed to pressure, and give generally a kindlier disposition to the entire process, and not unfrequently procure a speedy and happy termination to all its anxiety and peril."

Dr. Davis next proceeds to consider the rule generally maintained, at least, by English systematic writers, that it can never be necessary to have recourse to instruments as long as there is any positive progress made by the labour. While admitting this to be a good general rule, Dr. Davis contends that it is not without exceptions, and he adduces a case in illustration of this opinion, which, however, appears to us, to be calculated to afford it a very equivocal support. A lady, thirty years of age, was delivered of a still-born child, after a severe labour of eighteen hours, during no stage of which, could it be truly said that there was not some progress made. In a few hours from its commencement, the head began to enter the cavity of the pelvis, and was passed through, to use the author's impressive language, "in the midst of such a tempest of struggles as I think I have never witnessed upon any other occasion." There was nothing approaching to suspension of the action of the uterus, or arrest of the child's head. It was thought necessary to prescribe venesection about twelve hours after the commencement of the pains. The operation was proposed to be repeated about four hours afterwards, but unfortunately, this proposal was over-ruled. Such was the extreme irritation at that period that, in twenty minutes afterwards, the patient suffered a severe rigor of upwards of half an hour's duration. The child's head was by this time bearing strongly on the perineum, and the labour-pains were neither suspended nor perceptibly enfeebled, till the delivery was completed. The patient died on the tenth day after her delivery, and, on inspection of the body, a large abscess was found involving all the structures at the superior and left parts of the pelvis, of which the left ovarium appeared to have been the nucleus. Now, with all the deference due to Dr. Davis's better judgment, this case seems to us, by no means, to afford conclusive proof that the progressiveness of a labour is not a good and sufficient reason for abstaining from the use of instruments. Dr. Davis does not explain whether, in his opinion, the forceps ought to have been had recourse to, or whether he thinks it might not have been expedient and justifiable to diminish the size of the child's head, for the relief of the mother; and we are strongly inclined to believe that, if the suggestions which he made had been attended to, or if happily he had been left to manage the case, upon the principles which he so ably advocates in the present work, the labour might have been conducted to a safe conclusion, without the necessity of instrumental aid.

When there was no deficiency of parturient power, and while the gradual progress of the head shewed that there was no insurmountable deficiency of space, we cannot suppose that the danger and ultimate mischief could have arisen, except from the undue violence of the propellent force being disproportioned to the progress of relaxation of the soft parts, and from excessive excitement of the heart and arteries, both of which we submit, might have been so far moderated by general bleeding suitably to the exigency of the circumstances, combined with the judicious use of opium, as to have admitted of the labour being conducted to a safe termination, without artificial assistance.

On the use of instruments, on account of malposition of the child's head within the pelvis, Dr. Davis states the varieties of malposition liable to occur, but refers the consideration of the management suited to each to a subsequent part of the work, and then proceeds to speak of the use of instruments on account of hæmorrhage. On this subject, he describes the circumstances in which the operation of turning, and delivery by the forceps, should be respectively preferred. In the former operation, Dr. Davis makes the following practical observation.

"It is a fact, which it must have occurred to practitioners of experience to observe, that the operation of turning is, for the most part, speedily followed by a cessation of the hæmorrhage. The change thus effected in the situation of the child in utero being made, it is generally both unnecessary and improper to proceed hastily to complete the delivery, which, therefore, it would be much better to delay, for an hour or two, to wait for a favourable disposition of the soft parts, than to undertake immediately after having brought down the feet into the birth."

Dr. Davis adds his opinion, in which we fully concur, that

"The operation of turning would prove the means of saving a much greater number of children, without in any degree compromising the interests of the mother, than it really does, were it generally the practice to observe, on this very essentially practical point, the cautious forbearance here recommended."

The feet being once brought down into the vagina, it is a usual practice, by pulling at them, to bring the thorax or even the head, into the brim of the pelvis, in which situation the umbilical cord is liable to compression, and it is not in all cases that, even after the operation of turning has been effected, the head can be made to pass through the pelvis in so short a space of time as not to involve the child's life in jeopardy from this cause. By waiting a sufficient length of time after the feet are brought down, the soft parts become effectually relaxed, so that, when the parturient action of the uterus again acquires force, the head is made to follow the trunk with comparatively very little delay.

After pointing out the circumstances more especially demanding the use of the forceps, in cases of uterine hæmorrhage, our author concludes with remarking, that it is a caution almost inseparable from the use of the forceps, to apply it with perfect gentleness and dexterity, and to proceed *very slowly*; whereas, when its use is demanded, on account of profuse hæmorrhage, we are, of course, impressed with the necessity of expedition, and quick movements.

Rupture of the uterus and puerperal convulsions are not deemed, by Dr. Davis, as always indicating the immediate application of the forceps. When the former is spontaneous, it is generally connected with confinement of pelvis, and therefore, in cases not favourably suited for the use of that instrument. With respect to the opinion generally prevailing in the profession, that the accession of convulsions, during labour, forms an urgent indication for immediate delivery, and even for the use of instruments, if necessary to promote that object, Dr. Davis declares that he scarcely knows any single doctrine, in practical midwifery, more liable to serious objection. He says,

"I do not recollect a single instance of the accession of such convulsions, either immediately before, or at an early period of a labour, which had not been preceded by severe head-ache, great sense of weight in the head, giddiness, violent throbbing of the temporal arteries, impaired vision, or some other unequivocal symptom of an excessive fullness of the blood-vessels of the brain."

"Hence," again he observes, "the unusual turgescence, and visible throbbing of the great blood-vessels of the neck, the swelling and the intensely florid, and sometimes even the purple hue of the countenance; the rapid movements, wild expression, and, eventually, the vacant stare of the eyes, prominent and glistening, and as if ready to burst from their sockets on account of excessive distention of their blood-vessels; to which we may be allowed, in most cases, to add, an excruciating head-ache, and, not unfrequently, a delirious wandering of the understanding. Moreover, it is well known that the ordinary paroxysms of these convulsions are usually followed by coma, abolition of sense, stertorous breathing, and all the usual characteristic symptoms of a profound apoplexy, indicating, as in all other cases of apoplexy, the existence of dangerous pressure on the brain."

Acting upon this view of the proximate cause of puerperal convulsions, Dr. Davis is of opinion that the principal indication of treatment should have for its object, an effectual reduction of the pressure upon the brain, by the abstraction of blood, before resorting to the use of instruments.

For the descent of the umbilical cord, our author proposes two methods of treatment. The first is, to deliver with forceps with a narrow blade, on the side where the cord comes down, or with a short blade on that side, to act as a counterpart or fulcrum to one of his common broad blades. The second method is only applicable before the head has descended deeply into the pelvis. He proposes to take a very thin plate of elastic steel, like a spatula, fitted into a small wooden handle, and slightly curved, to correspond with the convexity of the child's head. Near the point of the instrument, there should be several small apertures, through which a thread being passed, the central part of the descending loop of cord might be fastened by its membranous coverings to the point of the instrument, which is then to be pushed up into the uterus, or, at least, above the child's head, and to be kept there, without withdrawing the instrument, till the head should have passed the os externum. Five, or even more, of such small spatulæ might, in the author's opinion, be used at once, if required, to keep up a large descent of cord.

In treating of syncope during labour, Dr. Davis introduces several very interesting cases for which, however, we are under the necessity of referring our readers to the work itself. His practical rules for such cases are summed up in few words.

When the head is high up, the orifice of the uterus not completely developed, and the pains languid or wholly suspended, the delivery should be effected by turning.

When the head is so far advanced into the pelvis as to make it very improbable that turning could be effected without exposing the patient to very considerable irritation, the liquor amnii having escaped, and the os uteri not sufficiently dilated to warrant an attempt to deliver with the forceps, cephalotomy and delivery with the crotchet must be had recourse to.

When the head is so low as to be within the reach of the forceps, and the state of the soft parts, such as to admit of its safe application, of course the forceps ought to be preferred.

Dr. Davis treats at considerable length, and under a distinct head, the importance of a just prognosis, in cases of midwifery where instruments are employed. His observations on this subject appear to us to be judicious and well calculated to repress that violent fondness for using instruments by which young practitioners, anxious to distinguish themselves, are sometimes apt to be led into dangerous errors. Speaking of the forceps in particular, our author, after reprobating, in severe terms, the habit which some persons have of boasting of the success with which they always wield that instrument, says, that in all cases, where the forceps is used, the parts are more or less liable to contusion. All the fangs and frame-work of the instrument are made of tempered steel; and, let them be ever so well covered and defended, they will still retain an unresisting hardness, calculated to bruize and fret any soft and living texture that might be interposed between their convex surfaces and the solid walls of the pelvis. These observations may be considered as applicable to the average of forceps cases, and without any special reference to such as are supposed to be involved in any extraordinary danger, excepting what might arise from the use of such an instrument. To the caution so judiciously inculcated in these observations of the learned author, we would only beg to add, that youthful practitioners should ever bear in mind, that it is not the mere fact of having used instruments, but the having done so to the effectual advantage of the patient, that can add to their reputation: and, whenever it becomes their duty to decide upon the necessity of employing instruments, they should not forget to ask themselves, whether, if the patient were the sister or wife of their affections, they would wish the same mode of treatment to be adopted for her recovery.

When the forceps or other obstetric instruments are to be used, the probability of a favourable issue must, of course, mainly depend upon the peculiar circumstances indicative of their use in each individual case. In rupture of the uterus, puerperal convulsions, and uterine hæmorrhage, the prognosis, as to the result to be anticipated from a resort to the use of the forceps, must of course be unfavourable. In cases of protracted or difficult labour from confinement of pelvis, or malposition of the child, the danger will be proportioned to the length of time the parts concerned have been exposed to pressure, from the fruitless exertion of the natural efforts, and other circumstances peculiar to each case. Dr. Davis's extended observations on this part of his subject are exceedingly well calculated to assist his readers in appreciating and justly estimating the value of each of the circumstances that can generally be considered as belonging to the elements of a safe prognosis.

Dr. Davis makes some brief but essential observations on the best position for the subject of an obstetric operation, and on the duty of an accurate examination per vaginam, and then proceeds to treat of the application of the short or common forceps, laying down his rules in numerical order, each accompanied with suitable comments and explanations. These rules require, 1st. That the head shall have reached a considerable depth below the brim of the pelvis: 2nd. That the blades be applied over the sides of the head and face: 3rd. That the forceps never be used till the orifice of the uterus shall have been amply dilated: 4th. That the left hand counterpart of the instrument, or that which is introduced at the left or undermost side of the pelvis be the first introduced: 5th. That the locking of the two blades never be effected with violence: 6th. That operations with the forceps always be very slowly performed: and 7th. That the force to be used never should exceed very moderate limits.

Dr. Davis gives ample directions, which, by the help of the plates, are rendered perfectly clear, as to the best mode of introducing the forceps. In two or three important particulars, at least, his instructions are essentially different from those usually insisted upon by preceding writers on midwifery.

"I have sometimes seen it recommended by authors," he observes, "to pass the conducting hand a great way up into the pelvis, under the pretence of making room for the easier introduction of the instrument.

But from the inconvenience generally of this method, if I may be allowed to speak from my own experience, from the actual obstruction given by the fingers to the easy introduction of the instrument; and from the liability of the fingers to be pinched by the convex surfaces of the claws of the forceps, I very much suspect that not only the rule itself is essentially incorrect in principle, but also that, in point of fact, it has seldom been observed in practice. The only proper use of the conducting fingers, is to direct accurately the point of the instrument, so as to insure its transmission to the side or other intended part of the child's head, without pinching, distending, or otherwise unnecessarily irritating the soft parts situated at the outlet of the pelvis. From this part of the head, the instrument is then to be passed gently along its left lateral surfaces, in a line somewhat lower in the pelvis than the known situation of the ear, until it is felt to have effected its transit over the temple and forehead. It should be gradually, and with great gentleness, raised up, and applied over the child's ear, so as to embrace easily, and at many points of its concavity, the corresponding convexity of the fœtal head. The entire blade is then to be moved upwards along the side of the pelvis, so as to be made to embrace accurately, within its fenestra, the left parietal protuberance, together with the greater part of the ear and side of the face, even to the chin."

To execute these movements accurately, it is necessary to pay particular attention to the situation of the ear relatively to the pelvis, before they are commenced. The introduction of the first blade is seldom attended with difficulty, and it should scarcely cause either inconvenience or pain to the patient. The other counter part of the forceps is usually called the right-hand blade, from being generally passed up with the right hand, and on the right side of the pelvis, the left hand being then used as a conductor. The common rule for the introduction of this blade directs, that it should be passed up as nearly as possible in the same direction relatively to the right side of the head, as the other branch had been to the left. To obey this rule would be extremely difficult, if not impracticable, unless the part to be operated upon were brought to project two or three inches over the side of the head. Without insisting upon this as an insuperable objection, Dr. Davis thinks it sufficient to warrant a departure from the prescribed rule, and this may be done without prejudice to the patients in either of two ways. The first, is to have the instrument made with a hinge or joint in the shank, so that the handle may fold out of the way of the bed. It has already been mentioned that Dr. Davis's, and the Edinburgh forceps, are furnished with contrivances of this kind.

But the introduction of the second branch may even be accomplished easily and advantageously without this contrivance, or without having the blade separable from the handle, as some have suggested.

"The left-hand blade being introduced and properly applied to the head, let its handle be moved backwards towards the inferior fourchette, to make way for the introduction of the practitioner's two first fingers of the left hand, which are now to be the conductors of the second or right-hand blade. These two fingers are to be passed into the vagina obliquely over the shank, and anteriorly to the handle of the branch of the instrument already introduced, so as to reach and be applied to the superior and posterior part of the child's head, an inch or two beyond the small fontanelle, in the direction of the sagittal suture.—The second branch of the forceps is then to be be taken up with the right hand, applied to, and gently carried up along the palm of the left, or conducting hand, into the vulva, so that its anterior surface, at and near the point, shall reach and be applied to the fœtal head, intermediately between the small fontanelle and the points of the two fingers of the operator's left hand." (Here the author refers to pl. iii. as beautifully illustrating his description.) "The point of the instrument having been introduced carefully, and without prejudice to the soft parts at the outlet of the pelvis, into the posterior part of the vagina; it must next be carried forwards, by an adroit circuitous movement, over the right parietal, coronal, and temporal regions of the head to its ultimate destination, and of course into easy and perfect parallelism with its antagonist blade."

(Here the author refers to pl. iv. as representing the blade by dotted lines in different stages of this movement.)

"The route taken during this movement of the instrument relatively to the pelvis, should correspond with a curved line drawn from the anterior and lateral part of the perinæum, obliquely across the posterior portions of the right sacro-ischiatic ligaments, and then gradually upwards to the right extremity of the transverse diameter of the brim of the pelvis; which of course will eventually bring it into strict parallelism, and more or less easy adjustment with its antagonist."

A view of the plates referred to, is sufficient to render these verbal descriptions perfectly perspicuous.

It sometimes happens, that from some defect in the form of the pelvis, affecting one part or side of it more than another, or, from a tumour occupying and narrowing one side of it, or from the umbilical cord descending on one side, it may be rendered extremely difficult to introduce two broad blades so as to admit of being easily, and without force, adjusted at the lock. Whenever such circumstances occur, they must be considered as diminishing the chance of ultimate success, as far as relates to the life of the child. Heretofore, in such untoward circumstances, when the natural powers were inadequate to the delivery, the attempt to deliver with the common forceps must either have been abandoned for cephalotomy, when the condition and safety of the mother imperiously required an early delivery, or the head must have been extracted at all events, and with great danger both to the mother and child with the forceps forcibly adjusted. To obviate these difficulties and dangers, our author recommends to the notice of the profession varieties of forceps, with blades of unequal length and breadth. In such cases of difficulty as above alluded to, one or other of these varieties may often be advantageously substituted for one of the broad blades, and serve as a sufficient fulcrum or antagonist to the other, without the least risk of the evils that might have been inseparable from using the two broad blades. For the particular description of these new and manifestly valuable additions to the mechanical powers of the art, we must again refer to the work itself and to the plates.

With respect to the rule generally insisted upon by all English, and most foreign authors on midwifery, that, when the forceps has once been introduced, it must never be withdrawn until the head of the child shall have been extracted, Dr. Davis expresses his decided dissent from the principle upon which it is founded.

"Nothing" he observes, "can be more absurd and unnecessary, not to say mischievous, than its universal, or even general adoption. In nine out of ten forceps cases, very moderate assistance, either slightly to change the position of the head, or to advance its progress beyond the seat of its temporary lodgement, as also beyond the operation of the impediment, which may have occasioned its arrest, is generally all that is required; so far am I from approving of the rule in question, that, in my own practice, I not only reject it, but almost always reverse it."

Again in the same paragraph he says,

"It is impossible to use an artificial power with so much admirable moderation and patience, as Nature herself exhibits in her usual very slow development of the maternal organs. But, if it be assumed that the instrument is not to be effectively used, whilst allowed to remain within the pelvis, beyond the actual demand for its proper power, and not at all after the extension of a certain amount of assistance, excepting on the failure of the natural efforts; then, I contend that it should be entirely withdrawn until such failure shall have declared itself."

The author gives detailed instructions for the use of the forceps in the first and second positions of the child's head, that is, with the forehead towards the sacrum and pubes respectively, recommending uniformly the utmost caution and gentleness; and, insisting strongly on the fact, that a very small degree of extracting force is generally sufficient to overcome all the difficulties of any simple forceps case.

"It is probable" says he, "that limited degrees of malposition of the fœtal head in the pelvis, should be considered as among the most frequent causes of protracted labours, such as are ultimately attended with the death of the child, but seldom succeeded by any bad consequences to the mother. A competent master of the art might often have it in his power to save the lives of children thus circumstanced, by the slightest possible application of a well adapted mechanical power. The required movement being once effected, nature would then prove quite equal to her proper work, and the demand for artificial aid would instantly cease."

"The final exit of the child's head should never be hurried; nor even allowed, under circumstances of instrumental interference, to take place with great rapidity. The necessary development of the inferior part of the vagina, including the perinæum and os externum, is almost always, and especially in first labours, the work of a long time. When Nature herself departs from her own proper law of effecting this part of the function very slowly, and by degrees scarcely perceptible, the structures more immediately concerned in the latter efforts of the struggle are liable to be torn, and are, it is known, sometimes actually lacerated."

In treating of the mode of operating with the forceps when the head of the child is in the third and fourth positions, that is, with the forehead towards the right and left sides of the pelvis respectively, our author is of opinion that its further descent is prevented from its long diameter not corresponding with the long diameter of the outlet of the pelvis; and the object of using the forceps is not so much to extract the head as to rectify its position by turning the face, if possible, into the hollow of the sacrum, or if this cannot be done, by turning the occipital vertex of the head towards the sacrum, so as to convert the presentation into one of the first or second position. In using the common forceps for this purpose there is always some difficulty and considerable risk of seriously injuring the bladder and other parts in front of the pelvis, as it is necessary to introduce one blade of the forceps between the head and symphysis pubis, towards which, one of the ears is turned in all cases of this kind. To obviate the risk attendant on the operation, and to diminish the difficulty of performing it, Dr. Davis has contrived a pair, or rather two pair of forceps, specially applicable to cases of the third and fourth positions. When the face is towards the right side of the pelvis, the short blade belonging to the forceps of the third position is to be introduced into the left sacro-iliac district of the pelvis, and applied to the latero-occipital region of the head, *i. e.* immediately behind the left ear, which part will be found to correspond with the left sacro-iliac junction of the pelvis. The long branch is then to be passed up at the right lateral and anterior region of the pelvis, so as to include within its fenestra, part of the right cheek and temple above and anteriorly to the right ear. The blades are then easily adjusted at the lock, and by a gentle rotatory movement of the head, the forehead is readily turned from the right, or upper side of the pelvis, into the hollow of the sacrum, and, of course, the occipital vertex will then present, in its natural position, under the arch of the pubes—this being effected the instrument is to be withdrawn, being no further required.

Cases of the fourth position, in which the face is directed towards the left side of the pelvis are to be treated upon the same principle, but with the instrument specially made for them.

It probably will be objected, that the proposed multiplication of instruments must lead to great inconvenience. To this, the reply must be, that the facilities obtained from operating with this variety of forceps, and the risk of delay, pain and worse consequences to the patient, which it enables us to avoid, will be found greatly to over-balance the inconvenience of being obliged to provide ourselves with it. This, at least, is the opinion we have formed of it after many comparative trials with it and the common forceps. In the machines in Dr. Davis's theatre, one circumstance that ought to weigh greatly in favour of the instrument is, that no part of it requires to be brought into contact either with the bladder or urethra during any part of the operation, a circumstance that alone decides its superiority over every other mode of operating that has heretofore been practised in cases of this kind.

With this remark, we take leave for the present of the subject, and of Dr. Davis's work, reserving to a future occasion the remarks we have to offer on the three remaining and very important sections, relating respectively to the "long forceps," and "other expedients for preserving the lives both of the mother and her offspring," and to "obstetric operations calculated to ensure the preservation of the more important life of the mother."

